# Effect of endogenous sodium and potassium ions in plants on the quality of alfalfa silage and bacterial community stability during fermentation

**DOI:** 10.3389/fpls.2023.1295114

**Published:** 2023-12-27

**Authors:** Jinhong Shi, Guijie Zhang, Wencan Ke, Yongxiang Pan, Meiling Hou, Chun Chang, Duowen Sa, Mingju Lv, Yinghao Liu, Qiang Lu

**Affiliations:** ^1^ College of Forestry and Prataculture, Ningxia University, Yinchuan, China; ^2^ College of Life Science, Baicheng Normal University, Baicheng, China; ^3^ Institute of Grassland Research, Chinese Academy of Agricultural Sciences, Hohhot, China; ^4^ Inner Mongolia Agriculture and Animal Husbandry Extension Center, Hohhot, China

**Keywords:** salt stress, endogenous ions, anaerobic fermentation, fermentation quality, microbial community

## Abstract

This study investigated the impact of endogenous sodium and potassium ions in plants on the quality of alfalfa silage, as well as the stability of bacterial communities during fermentation. Silage was produced from the fermented alfalfa, and the chemical composition, fermentation characteristics, and microbiome were analyzed to understand their interplay and impact on silage fermentation quality. The alfalfa was cultivated under salt stress with the following: (a) soil content of <1‰ (CK); (b) 1‰–2‰ (LP); (c) 2‰–3‰ (MP); (d) 3‰–4‰ (HP). The results revealed that the pH of silage was negatively correlated with the lactic acid content. With the increase of lactic acid (LA) content increased (26.3–51.0 g/kg DM), the pH value decreased (4.9–5.3). With the increase of salt stress, the content of Na^+^ in silage increased (2.2–5.4 g/kg DM). The presence of endogenous Na^+^ and K^+^ ions in plants significantly affected the quality of alfalfa silage and the dynamics of bacterial communities during fermentation. Increased salt stress led to changes in microbial composition, with *Lactococcus* and *Pantoea* showing a gradual increase in abundance, especially under high salt stress. Low pH inhibited the growth of certain bacterial genera, such as *Pantoea* and *Pediococcus*. The abundance of *Escherichia*–*Shigella* and *Comamonas* negatively correlated with crude protein (CP) content, while *Enterococcus* and *Lactococcus* exhibited a positive correlation. Furthermore, the accumulation of endogenous Na^+^ in alfalfa under salt stress suppressed bacterial proliferation, thereby reducing protein degradation during fermentation. The pH of the silage was high, and the LA content was also high. Silages from alfalfa under higher salt stress had higher Na^+^ content. The alpha diversity of bacterial communities in alfalfa silages showed distinct patterns. Desirable genera like *Lactococcus* and Lactobacillus predominated in silages produced from alfalfa under salt stress, resulting in better fermentation quality.

## Introduction

According to statistics from the United Nations Educational, Scientific and Cultural Organization (UNESCO), saline–alkali soils are widely distributed worldwide, spanning more than 100 countries and encompassing roughly 25% of the global land area (Yun et al., 2023). In China, the total extent of saline–alkali soils is approximately 33.51 million hectares, accounting for 4.88% of the country’s total land area. These soils are predominantly found in the North China, Northeast, and Northwest inland regions, with approximately 30% being agriculturally viable (Li and Wang, 2018). A prominent feature of saline–alkali soils is the excessive accumulation of Na^+^ and K^+^ ions. The presence of Na^+^ has a profound impact on the physical, chemical, and biological properties of the soil (Hanum et al., 2022). Consequently, these factors contribute to soil structural instability, deterioration of soil hydraulic characteristics, nutrient imbalances in plants, and diminished vegetation coverage (Zhao et al., 2018).

Alfalfa (*Medicago sativa* L.) is an excellent leguminous forage characterized by its high nutritional content and palatability to livestock, alfalfa has a deep root system and strong nitrogen-fixing capacity, making it highly valuable for soil improvement (Xie et al., 2023). In saline–alkali soils, due to the superior nutrient quality, alfalfa can provide substantial quantities of high-quality protein, with silage being one of the primary preservation and utilization methods, particularly in environments with limited availability such as the rainy season. In recent years, research has shown that Na^+^ and K^+^ in plants have a significant impact on the quality of silage and the microbial dynamics during fermentation (Qiang et al., 2021b).

Ensiling is a fermentation process in which lactic acid bacteria (LAB), convert into lactic acid (LA) and other organic acids, thereby reducing the pH of the ensiled feed and inhibiting the growth of harmful bacteria, thus preserving the nutritional components of the silage (Nazar et al., 2020). However, silage fermentation is a complex process involving multiple microbial interactions, and the types and quantities of microorganisms directly influence the quality of silage. Salt is considered to be one of the critical factors affecting microbial growth and metabolism during fermentation. It is worth noting that the toxicity of Na^+^ is higher than that of Cl^−^, and an increase in Na^+^ concentration can inhibit microbial activity and interfere with their metabolism (Ye et al., 2008).

The concentration of salt has been found to influence the microbial community during fermentation (Jeong et al., 2021). According to Yang et al. (2020), their study on the fermentation of Northeast-style sauerkraut at different salt concentrations revealed that the dominant genera were *Lactobacillus* and *Leuconostoc*. In sauerkraut samples with 0.5% salt, Lactobacillus was the most abundant genus, accounting for 88.46% of the total. The population of Lactobacillus gradually increased in samples with 0.5% salinity but showed a decreasing trend in samples with the three salt concentrations (1.5%, 2.5%, and 3.5%). These findings indicate that salt concentration significantly affects the microbial community. According to Sarkar et al. (2020), a low level of NaCl improves hydrolysis and acidification. Huang et al. (2022) conducted a study on acid production during fermentation of high-salt kitchen wastewater. They demonstrated the trend of diminishing acid production by LAB with an upsurge in NaCl concentration from 0 to 8 g/L. While optimal concentrations of Na^+^ can indeed enhance enzymatic activities and help maintain osmotic balance, an excess can suppress LAB growth and fermentative function.

While the biotechnological applications of LAB have been extensively investigated in various contexts, their specific roles and potential still require meticulous research. To seek the better fermentation effect as we provided, this study hypothesizes that the endogenous levels of Na^+^ and potassium K^+^ in alfalfa plants are pivotal in determining the quality of alfalfa silage and the stability of the bacterial community during fermentation. We propose that optimal concentrations of these ions will enhance fermentation quality by supporting the growth and metabolic activity of beneficial lactic acid bacteria. In contrast, deviations from these optimal levels may impair silage quality and disrupt microbial homeostasis. Ultimately, this research may contribute to the development of strategies to enhance the nutritional value and microbial stability of alfalfa silage.

## Material and methods

### Experimental field and preparation of silage

The experiment was carried out at the Baotou Experimental Station for Forage Processing and High Efficient Utilization, located in Baotou City, Inner Mongolia, China. This region, situated in the Hetao Plain, is known for its high salinity. Geographically, the experimental site spans between 110°37”∼110°27” E and 40°05”– 40°17” N. The climate of the area is characterized as a north-temperate continental climate with arid and windy conditions. The prevailing wind direction throughout the year was northwest. The average annual temperature is 6.8 °C, and the frost-free period lasts approximately 165 days. Annual average rainfall measures 330 mm, while the average evaporation rate is 2094 mm.

### Description of raw materials and preparation of silage

The field experiments were conducted in 2022 utilizing the ZhongMu No.3 variety of alfalfa, provided by the Beijing Institute of Animal Science and Veterinary Medicine of the Chinese Academy of Agricultural Sciences. This particular variety of alfalfa exhibited strong salt resistance, excellent palatability, high nutritional value, and richness. In May 2022, alfalfa was sown using a drilling method with a row-to-row distance of 10 cm. Four different positions were selected to represent varying levels of salt stress: non-stress (CK), low stress (LP), moderate stress (MP), and high stress (HP). The salt stress contents at the CK, LP, MP, and HP sites were <1‰, 1‰–2‰, 2‰–3‰, and 3‰–4‰, respectively. Each group was replicated three times. The physical properties of the soil are presented in **Table 1** for reference.

**Table 1:** The physical and chemical properties of soils.

Indictors	Na^+^(g/kg)	K^+^(g/kg)	pH	EC (mS/cm)
CK	0.11± 0.006c	0.027 ± 0.001d	7.4 ± 0.21b	0.21 ± 0.02a
LP	0.15 ± 0.004b	0.031 ± 0.001c	8.4 ± 0.08a	0.59 ± 0.10b
MP	0.16 ± 0.002b	0.035 ± 0.001b	8.6 ± 0.11a	1.35 ± 0.01c
HP	0.25 ± 0.019a	0.041 ± 0.001a	8.7 ± 0.34a	2.3 ± 0.29d

CK, without salt stress; LP, under light salt stress; MP, under moderate salt stress; HP, under severe salt stress; EC, electrical conductivity. Numbers in a column followed by different lowercase letters differ at P < 0.05.

Alfalfa was harvested in the initial flowering stage. Then, wilted for 5 hours to obtain a targeted dry matter (DM) content, and immediately chopped into 2–3 cm lengths by a fodder chopper. Each material was treated separately to prevent crossing contaminations. 2 kg of the prepared alfalfa were packed in polyethylene plastic bags and sealed with a vacuum sealer in each group. All bags were assigned without additives. To investigate the effect of endogenous Na^+^ and K^+^ ions in plants on the quality of alfalfa silage and bacterial community stability during fermentation, triplicate samples for each group were prepared. Triplicate for each group was opened after 1, 3, 5, 7, 15, and 30 days of ensiling, respectively.

### Chemical composition and organic acid

The DM of the fresh alfalfa and silage was determined by oven drying at 65°C for 72 h. The neutral detergent fiber (NDF) and acid detergent fiber (ADF) were measured according to Van Soest’s procedures (Van et al., 1991). Colorimetry after reaction with anthrone reagent was used to determine the starch and water-soluble carbohydrate (WSC) content (Qiang et al., 2021b). Non-structural carbohydrates (NSC) are the sum of WSC and starch. The crude protein (CP = total N × 6.25) was determined using a Kjeldahl apparatus (Gerhart Vapodest 50 s, Germany) and the soluble protein (SP) was performed using the trichloroacetic acid method according to Cunniff (1997). The concentrations of Na^+^ and K^+^ ions of alfalfa were measured relative to standard solutions using a model 425 flame photometer (Sherwood Scientific Ltd., UK).

To assess the fermentation characteristics of the forage, 10 g of silage was mixed with 90 g of deionized water. The liquid extract was filtered through four layers of cheesecloth and filtered paper. The prepared filtrates were determined for measuring pH, ammonia nitrogen (ammonia-N), and organic acids. The pH was measured immediately with a glass electrode pH meter (LEICI pH S-3C, Shanghai, China). The content of ammonia-N was followed by the phenol-hypochlorite procedure of Broderick and Kang (1980). The concentration of organic acids were determined by high-performance liquid chromatography (HPLC, Waters e2695, Massachusetts USA; column: Waters Symmetry C18; oven temperature, 50°C; mobile phase 3 mmol L^–1^ perchlorate solution; flow rate 1.0 mL min^–1^; flame photometric detector 210 nm; sample size 5.0 μL) of Ping et al. (2017). Buffering capacity (BC) was determined by the hydrochloric acid sodium hydroxide method (Lin et al., 1992).

### Microorganisms enumeration

Microbial enumeration was performed using a 10 g fresh sample or silage. The sample was shaken with 90 mL of sterile distilled water at 120 rpm for 2 hours. From this solution, 1 mL was extracted and subjected to a 10-fold serial dilution for microorganism enumeration. The remaining solution was filtered and stored in a −80°C refrigerator for DNA extraction. Enumeration of LAB colonies was conducted on MRS agar medium (Nissui seiyaku Ltd., Tokyo, Japan). The plates were incubated in an anaerobic incubator (Heal Force Instrument Manufacturing Co., Ltd., Shanghai, China) at 37°C for 48 hours. Aerobic bacteria were cultured and counted on a nutrient agar medium, while yeasts were counted on potato dextrose agar (Nissui-seiyaku Ltd., Tokyo, Japan). Enumeration of *Enterobacteriaceae* was performed on Violet Red Bile Glucose Agar medium under aerobic conditions after 48 hours of incubation at 37°C. Colony-forming units (cfu) were used to express the microbial data, which were further transformed to a logarithmic scale on a fresh matter (FM) basis.

### High throughput sequencing of microbial population

The E.Z.N.A.^®^ Plant DNA Kit (Omega Bio-Tek, Norcross, GA, U.S.) was employed to extract microbial DNA from alfalfa samples, following the manufacturer’s protocols. The concentration and purity of the final DNA were assessed using a NanoDrop 2000 UV-vis spectrophotometer (Thermo Scientific, Wilmington, USA), and the quality was verified through 1% agarose gel electrophoresis. To amplify the V3–V4 hypervariable regions of the bacteria 16S rRNA gene, a thermocycler PCR system (GeneAmp 9700, ABI, USA) was utilized with primers 338F (5’-ACTCCTACGGGAGGCAGCAG-3’) and 806R (5’-GGACTACHVGGGTWTCTAAT-3’). The PCR reactions consisted of an initial denaturation at 95°C for 3 minutes, followed by 27 cycles of denaturation at 95°C for 30 seconds, annealing at 55°C for 30 seconds, elongation at 72°C for 45 seconds, and a final extension at 72°C for 10 minutes. Each 20 μL reaction mixture included 4 μL of 5 × FastPfu Buffer, 2 μL of 2.5 mM dNTPs, 0.8 μL of each primer (5 μM), 0.4 μL of FastPfu Polymerase, and 10 ng of template DNA. The PCR reactions were performed in triplicate.

The resulting PCR products were extracted from a 2% agarose gel and purified using the AxyPrep DNA Gel Extraction Kit (Axygen Biosciences, Union City, CA, USA). Quantification was conducted using the QuantiFluor™-ST (Promega, USA) as per the manufacturer’s instructions. The raw fastq files underwent demultiplexing and quality filtering using Trimmomatic. Subsequently, they were merged using FLASH based on the following criteria: (a) reads with an average quality score <20 over a 50 bp sliding window were truncated; (b) primers were allowed up to 2 nucleotide mismatches, and reads containing ambiguous bases were discarded; (c) sequences with an overlap longer than 10 bp were merged. Operational taxonomic units (OTUs) were clustered at a 97% similarity cutoff using UPARSE, while UCHIME was employed for the identification and removal of chimeric sequences. The RDP Classifier algorithm was used to analyze the taxonomy of each 16S rRNA gene sequence against the Silva (SSU123) 16S rRNA database, with a confidence threshold of 70%.

### Statistical analysis

The statistical data were analyzed by the procedure of SAS (version 9.3, SAS Institute Inc., Cary, NC, USA). Duncan’s multiple range tests were used to evaluate differences among groups. High throughput sequencing data were performed using an online platform of Majorbio I-Sanger Cloud Platform (www.i-sanger.com).

## Results

### The chemical composition of fresh alfalfa

The chemical composition and microbial population of fresh alfalfa under various salinity groups are detailed in **Table 2**. It is evident that salt stress markedly affected the Na^+^ and K^+^ content in the alfalfa, with significant differences (*P* < 0.05). Specifically, the LP group resulted in the highest K^+^ concentration, at 31.2 g/kg (*P* < 0.05), while the highest Na^+^ concentration was recorded in the HP group, at 5.1 g/kg (*P* < 0.01). The DM content varied slightly between groups, ranging from 304.3 to 319.0 g/kg, with the MP group demonstrating the highest DM content. SP content was significantly greater in the MP group (103.7 g/kg) compared to other groups (*P* < 0.01). The WSC content was notably lower in the CK group (366.3 g/kg) than in other groups (*P* < 0.01). Furthermore, the alfalfa subjected to the HP group exhibited the highest NDF content (467.7 g/kg), while the lowest was observed in the LP group (420.3 g/kg), demonstrating a significant effect of salt stress on NDF (*P* < 0.01). Interestingly, the MP group had significantly lower counts of aerobic bacteria, only 6.95 Log_10_ cfu/g (*P* < 0.01), which underscores the influence of salt stress on the microbial populations associated with the plant.

**Table 2:** Chemical composition and microbial populations of fresh alfalfa.

Items	CK	LP	MP	HP	SEM	*P*-value
Dry matter (g/kg FM)	304.3	312.7	319.0	305.3	0.25	0.21
Crude protein (g/kg DM)	192.3C	207.8B	234.3A	207.0B	0.11	0.19
Soluble protein (g/kg DM)	66.3C	72.3B	103.7A	68.3BC	0.08	<0.05
Neutral detergent fiber(g/kg DM)	432.0B	420.3C	474.3A	467.7A	0.12	<0.05
Acid detergent fiber (g/kg DM)	353.7C	348.0D	386.3A	381.0B	0.07	<0.05
Water-soluble carbohydrate (g/kg DM)	366.3C	372.3B	394.0A	368.3BC	0.07	<0.05
Lactic acid bacteria (Log_10_ cfu/g FM)	6.59	6.42	6.49	6.41	0.03	0.23
*Enterobacteriaceae* (Log_10_ cfu/g FM)	4.41A	4.21B	4.30AB	4.19B	0.03	0.06
Mold (Log_10_ cfu/g FM)	5.59	5.53	5.49	5.36	0.08	0.77
Aerobic bacteria (Log_10_ cfu/g FM)	7.31A	7.03BC	6.95C	7.04B	0.01	<0.05
Na^+^ (g/kg DM)	1.9D	2.7C	4.8B	5.1A	0.40	<0.05
K^+^ (g/kg DM)	26.7D	31.2A	30.9B	29.4C	0.53	<0.05

CK, without salt stress; LP, under light salt stress; MP, under moderate salt stress; HP, under severe salt stress; DM, dry matter; FM, Fresh matter; cfu, colony forming unit; SEM, standard error of mean value. The mean values (a–d) of different letters in the same column were significantly different (P < 0.05).

### Fermentation characteristics and chemical composition of alfalfa silage

The fermentation characteristics of alfalfa silage at different days of ensiling are presented in **Table 3**. The results indicated that salt stress (T), ensiling days (D), and their interaction significantly (*P* < 0.01) affected the pH, LA, Butyric acid (BA), Acetic acid (AA), and ammonia nitrogen content. After 30 days of ensiling, the pH of alfalfa silage ranged from 4.93 to 5.12, with a significantly lower pH observed for MP silage compared to other silages (*P* < 0.05). The pH of silage at different fermentation stages within 1–30 days were significantly different (*P* < 0.05), with a gradual decrease in pH as the ensiling time increased, with HP silage decreasing from 6.23 on day 1 to 5.13 on day 30, which was a significant decrease of 1.1 of pH. The rapid decrease in pH (**Table 3**) led to an 80g/kg reduction in WSC content for 30-day MP silage compared to 1-day silage (384.3 g/kg vs. 304.3 g/kg, respectively, **Table 4**). The AA contents of the 30-day silage ranged from 31.4g/kg to 46.7g/kg, with significantly lower AA content observed for HP silage compared to other silages (*P* < 0.05). With an increase in salt stress, the accumulated amount of AA in silages for the first 15 days showed an increasing trend across all four groups, but a decreasing trend was observed for MP and HP silages on day 30. BA content ranged from 13.1g/kg to 16.4g/kg, with CK silage having the highest BA content (16.4g/kg) (*P* < 0.05) on day 30 of fermentation and the lowest LA content (26.3 g/kg) (*P* < 0.05). The ammonia nitrogen contents of CK and LP silages were significantly (*P* < 0.05) higher than that of MP and HP silages at 30 days of ensiling.

**Table 3:** Fermentation characteristics of alfalfa silage on different days of silage.

Items	Group	Ensiling(d)						SEM	*P*-value		
		1d	3d	5d	7d	15d	30d		T	D	T×D
pH	CK	6.20ABa	5.99ABb	5.73Ac	5.18Bd	5.18Ad	5.12Ad	0.01	<0.01	<0.01	<0.01
	LP	6.17BCa	5.90ABb	5.83Ab	4.91Cc	4.67Dd	4.99ABc				
	MP	6.13Ca	5.83Bb	5.24Cc	4.84Cde	4.76Ce	4.93Bd				
	HP	6.23Aa	6.03Ab	5.61Bc	5.43Ad	5.17Ae	5.13Ae				
Ammonia-N (g/kg DM)	CK	10.3Ae	12.4Ad	15.9Ac	18.7Ab	23.6Ba	24.3Ba	0.01	<0.01	<0.01	<0.01
	LP	8.7Bf	12.6Ae	14.7Bd	17.3Bc	24.3Ab	27.3Aa				
	MP	8.7Be	11.7Bd	14.1Cc	16.8Bb	17.1Cab	17.7Ca				
	HP	7.1Ce	10.8Cd	12.4Dc	14.4Cb	14.7Dab	15.4Da				
Lactic acid (g/kg DM)	CK	10.8Ae	15.6Ad	19.7Cc	23.1Bb	25.8Da	26.3Da	0.01	<0.01	<0.01	<0.01
	LP	10.5Af	16.1Ae	20.5Bd	28.1Ac	38.7Ab	45.6Aa				
	MP	10.2Af	14.5Be	21.9Ad	28.9Ac	36.6Bb	43.2Ba				
	HP	8.4Bf	13.6Ce	20.2BCd	23.9Bc	31.4Cb	40.1Ca				
Acetic acid (g/kg DM)	CK	3.3Af	12.4Ae	27.7Ad	31.5Ac	41.8Ab	46.7Aa	0.01	<0.01	<0.01	<0.01
	LP	3.6Af	11.3Be	24.5Bd	30.2Bc	38.4Bb	41.2Ba				
	MP	2.7Bf	11.4Be	23.0Cd	29.3Cc	37.5Ca	32.9Cb				
	HP	1.9Cf	10.5Ce	21.8Dd	29.6BCc	36.6Da	31.4Cb				
Butyric acid (g/kg DM)	CK	2.5Bf	5.6Ae	8.7Ad	12.5Ac	14.4Ab	16.4Aa	0.003	<0.01	<0.01	<0.01
	LP	3.1Af	5.6Ae	7.8Bd	9.6Cc	13.5Bb	15.4Aa				
	MP	201Ce	4.7Bd	6.7Cc	10.3Bb	12.5Ca	12.7Ba				
	HP	1.1Df	2.5Ce	4.7Dd	7.6Dc	09.5Db	13.1Ba				

CK, without salt stress; LP, under light salt stress; MP, under moderate salt stress; HP, under severe salt stress; DM, dry matter; T, salt stress; D, ensiling day; T × D, interaction between salt stress and silage days; SEM, standard error of mean value. The mean values (a–f) of different letters in the same column were significantly different (P < 0.05).

**Table 4:** Study on chemical composition of alfalfa silage on different days of silage.

ltems	Group	Ensiling(d)						SEM	*P*-value		
		1d	3d	5d	7d	15d	30d		T	D	T×D
Dry matter (g/kg FM)	CK	304.7Aa	293.3Aab	281.7Abc	275.7Bc	273.3Ac	270.7Ac	0.1	0.02	<0.01	0.11
	LP	305.3Aa	301.0Aa	298.3Aa	298.7Aa	277.0Ab	267.3Ab				
	MP	314.7Aa	292.0Aab	277.7Ab	283.7ABb	291.0Aab	280.3Ab				
	HP	303.7Aa	301.3Aa	288.0Aab	289.0ABab	288.0Aab	275.3Ab				
Crude protein (g/kg DM)	CK	203.7Ba	197.3Bab	191.7Cb	190.3Bbc	180.7Bd	181.3Ccd	0.05	<0.01	<0.01	<0.01
	LP	204.0Ba	198.0Bab	185.0BCabc	196.0Babc	187.7Bc	193.0Bbc				
	MP	228.7Aa	228.0Aa	230.0Aa	223.3Aa	215.7Ab	206.3Ac				
	HP	204.3Ba	203.3Ba	201.0Bab	196.7Bb	188.7Bc	195.3Bb				
Soluble protein (g/kg DM)	CK	66.7Df	73.7Ce	86.7Dd	99.7Cc	110.7Cb	132.7Ca	0.02	<0.01	<0.01	<0.01
	LP	75.7Bf	80.0Be	92.3Cd	98.7Cc	126.7Ab	150.3Aa				
	MP	104.3Af	108.3Ae	113.3Ad	116.7Ac	122.3Bb	124.3Da				
	HP	71.0Cf	82.3Be	94.7Bd	104.3Bc	126.3Ab	138.3Ba				
Neutral detergent fiber (g/kg DM)	CK	428.0Ca	423.3Cb	419.3Bc	417.7Bc	418.3Bc	407.7Bd	0.03	<0.01	<0.01	<0.01
	LP	421.7Da	417.7Da	408.7Cb	395.3Cc	383.7Cd	377.0Ce				
	MP	469.3Aa	462.3Ab	456.3Ac	447.3Ad	442.3Ae	439.0Af				
	HP	463.3Ba	459.7Bb	455.7Ac	449.3Ad	442.3Ae	436.30Af				
Acid detergent fiber (g/kg DM)	CK	351.3Ca	347.7Cb	341.0Cc	337.3Cd	335.7Ce	327.7Cf	0.02	<0.01	<0.01	<0.01
	LP	347.0Da	342.3Db	337.3Dc	332.3Dd	328.0De	325.3Df				
	MP	381.3Aa	375.3Ab	367.7Bc	361.3Bd	354.3Be	347.3Bf				
	HP	381.3Aa	377.0Ab	370.7Ac	366.7Ad	359.0Ae	350.7Af				
Water-soluble carbohydrates (g/kg DM)	CK	356.7Ca	353.7Ba	346.7Bb	336.3Bc	317.3Ad	312.7Ad	0.04	<0.01	<0.01	<0.01
	LP	365.7Ba	346.7Cb	339.0Bc	328.7Cd	316.7Ae	303.7Bf				
	MP	384.3Aa	378.3Aa	363.3Ab	343.3Ac	312.3ABd	304.3Bd				
	HP	354.3Ca	342.3Cb	334.7Bc	325.3Cd	309.7Be	305.0Be				

CK, without salt stress; LP, under light salt stress; MP, under moderate salt stress; HP, under severe salt stress; FM, Fresh matter; DM, dry matter; T, salt stress; D, ensiling day; T × D, interaction between salt stress and silage days; SEM, standard error of mean value. The mean values (a–f) of different letters in the same column were significantly different (P < 0.05).

The results showed that salt stress (T), ensiling days (D), and their interaction significantly (*P* < 0.01) affected the contents of WSC, CP, NDF, and ADF (**Table 4**). In the study, the NDF content ranged from 377g/kg to 439g/kg, and the SP content ranging from 124.3g/kg to 150.3g/kg. Both T and D significantly influenced the NDF and SP content (*P* < 0.05). After 30 days of ensiling, the SP content of the MP group (124.3 g/kg) was significantly lower than that of the other groups. The CP contents of 30-day silage ranged from 181.3g/kg to 206.3g/kg, with significantly higher CP content observed for MP silage compared to other silages (*P* < 0.05).

### Analysis of microbial community quantities

Analysis revealed significant effects (*P* < 0.01) of salt stress (T), ensiling days (D), and the interaction between salt stress and ensiling days on the quantities of LAB, *Escherichia coli*, molds, and general aerobic bacteria (**Table 5**). In terms of ensiling fermentation days, the quantity of LAB in alfalfa silage reached its peak after 3 days of fermentation and then leveled off. The CK silage group had a significantly higher quantity of LAB than the other groups, at 7.66 Log_10_ cfu/g. There were no significant differences (*P* > 0.05) in the quantity of LAB between the 5d, 7d, 15d, and 30d fermentation periods. At 1 day of alfalfa ensiling fermentation, the number of LAB in the CK group was significantly lower than in the other groups (*P* < 0.05), at 6.97 Log_10_ cfu/g. Overall, except for the 3d ensiling, the MP silage group had a significantly higher quantity of LAB than the other group (*P* < 0.05). There were no significant differences (*P* > 0.05) in the quantity of LAB between the 15d and 30d fermentation periods, indicating that the quantity of LAB in salt–alkali alfalfa silage reached a stable state after 30 days of fermentation.

**Table 5:** Study on microbial quantity of alfalfa silage on different days of silage.

Items	Groups	Ensiling(d)							*P*-value		
		1d	3d	5d	7d	15d	30d	SEM	T	D	T×D
Lactic acid bacteria (Log_10_ cfu/g FM)	CK	6.97Bd	7.66Aa	7.58Aab	7.56Aab	7.53Bbc	7.45Ac	0.01	<0.01	<0.01	<0.01
	LP	7.02Bb	7.61Aa	7.61Aa	7.52Aa	7.52Ba	7.57Aa				
	MP	7.19Ab	7.58Aa	7.65Aa	7.61Aa	7.68Aa	7.53Aa				
	HP	7.25Ac	7.45Ab	7.33Bc	7.51Aab	7.51Bab	7.59Aa				
*Enterobacteriaceae* (Log_10_ cfu/g FM)	CK	4.41Ba	3.10Ab	3.10Ab	ND	ND	ND	0.06	0.2	<0.01	<0.01
	LP	4.43Ba	2.26Ab	3.32Aab	ND	ND	ND				
	MP	6.17Aa	2.16Ab	ND	ND	ND	ND				
	HP	6.14Aa	3.16Ab	ND	ND	ND	ND				
Mold(Log_10_ cfu/g FM)	CK	6.01Aa	ND	ND	ND	ND	ND	0.01	<0.01	<0.01	<0.01
	LP	5.88Ba	ND	ND	ND	ND	ND				
	MP	ND	ND	ND	ND	ND	ND				
	HP	ND	ND	ND	ND	ND	ND				
Aerobic bacteria (Log_10_ cfu/g FM)	CK	7.31Aa	7.15Ab	7.09Ab	6.96Ac	7.05Abc	6.77Bd	0.004	<0.01	<0.01	<0.01
	LP	7.05Cb	7.15Aa	6.94Cc	6.98Ac	6.97Bc	6.82Bd				
	MP	7.15Ba	7.07Bb	7.02Bbc	6.99Ac	6.97Bc	6.78Bd				
	HP	7.01Ca	6.99Ca	6.94Cbc	6.97Aab	6.92Bc	6.92Ac				

CK, without salt stress; LP, under light salt stress; MP, under moderate salt stress; HP, under severe salt stress; ND, not detected; cfu, colony forming unit; T, salt stress; D, ensiling day; T × D, interaction between salt stress and silage days; SEM, standard error of mean value. The mean values (a–d) of different letters in the same column were significantly different (P < 0.05).

From the perspective of ensiling fermentation days, the quantity of *Escherichia coli* in alfalfa silage reached its peak after 1 day of fermentation, and then gradually decreased. The MP group (6.17 Log_10_ cfu/g) had a significantly higher quantity of *Escherichia coli* than the CK and LP groups. At 3 days of fermentation, the quantity of *Escherichia coli* in the MP silage group was significantly lower than in the other groups, at 2.16 Log_10_ cfu/g. *Escherichia coli* was not detected in the MP and HP groups at 5d of fermentation. *Escherichia coli* was not detected in any of the groups at 7d, 15d, and 30d of fermentation. From the perspective of ensiling fermentation days, the quantity of molds in alfalfa silage gradually decreased after 1d of fermentation, and no molds were detected at 3d, 5d, 7d, 15d, and 30d of fermentation. At 1 day of alfalfa silage fermentation, the quantity of molds in the CK group was significantly higher than in the LP group (*P* < 0.05), while no molds were detected in the MP and HP groups, indicating better preservation in the later stages.

In terms of ensiling fermentation days, the quantity of general aerobic bacteria in alfalfa silage reached its peak after 1 day of fermentation, and then gradually decreased. The CK group had a significantly higher quantity of general aerobic bacteria than the other groups (*P* < 0.05). There were significant differences (*P* < 0.05) in the quantity of general aerobic bacteria between the 5d, 7d, 15d, and 30d fermentation periods. At 30d of fermentation, the HP group had the highest quantity of general aerobic bacteria, at 6.92 Log_10_ cfu/g. However, the quantity of general aerobic bacteria was the lowest at 1d of fermentation, at 7.01 Log_10_ cfu/g.

### Analysis of bacterial community composition

**Figure 1:**
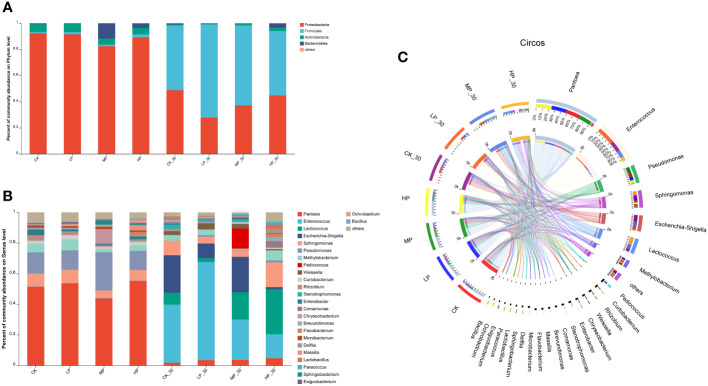
Bacterial communities and relative abundance by phylum level **(A, C)** and genus level **(B)** for raw alfalfa and alfalfa silage. CK, without salt stress; LP, under light salt stress; MP, under moderate salt stress; HP, under severe salt stress; 30, 30 days of ensiling.

To gain further insights into the dynamic succession of bacterial communities in alfalfa silage under salt stress, we assessed the bacterial communities at the phylum and genus levels (**Figure 1**). The bacterial communities in fresh alfalfa and silage feed were mainly composed of four phyla (**Figure 1A**). Before ensiling, the phylum Proteobacteria had the highest abundance, followed by Actinobacteria, Bacteroidetes, and Firmicutes. After ensiling, Firmicutes became the dominant phylum. Compared to the other groups, the MP-30 group showed a higher abundance of Firmicutes in the silage feed. At the genus level, the dominant genera in the pre-ensiling group were *Pediococcus*, *Pseudomonas*, and *Sphingomonas*. In the HP group, the relative abundance of *Pediococcus* and *Pseudomonas* was higher than in the other groups, while *Escherichia–Shigella* had a lower relative abundance. After 30 days of ensiling, the dominant genera in all groups were *Enterococcus*, *Lactococcus*, *Escherichia–Shigella*, and *Sphingomonas* (**Figure 1B**). *Enterococcus* and *Escherichia–Shigella* were the dominant genera in CK-30 and LP-30 silage feed. *Lactococcus* dominated in the HP-30 group. The lowest concentrations of *Pseudomonas* and *Flavobacterium* were observed in the MP-30-treated silage feed.

With prolonged fermentation time, the relative abundance of *Pantoea* gradually decreased, while *Enterococcus* and *Pseudomonas* gradually became dominant (**Figure 1C**). In the LP-30 silage, after establishing favorable anaerobic conditions, *Enterococcus* continued to proliferate, inhibiting the growth of LAB, leading to a gradual decrease in the abundance of *Lactococcus* and LAB, and an increase in the abundance of *Enterococcus*. Furthermore, both low and high levels of salt stress were unfavorable for the growth of *Escherichia–Shigella*.

**Figure 2:**
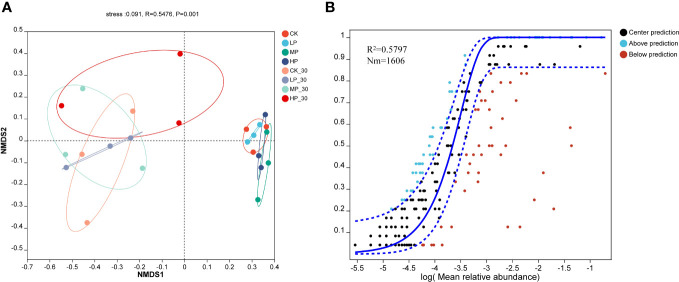
The non-metric multidimensional scaling (NMDS) on genus level for raw alfalfa and alfalfa silage **(A)** and the neutral community model (MCN) of silage on genus level **(B)**. CK, without salt stress; LP, under light salt stress; MP, under moderate salt stress; HP, under severe salt stress; 30, 30 days of ensiling.

The Non-metric Multidimensional Scaling (NMDS; **Figure 2A**) and Neutral Community Model (NCM; **Figure 2B**) analyses performed in this study reveal the variations in bacterial communities during anaerobic fermentation. The stress value of 0.091 indicates a good fit of the data to the model. The NMDS analysis demonstrates similarities and differences between the samples from different groups.

This diagram shows the relative positions of the samples in each of the eight groups. The HP group shows a significant difference compared to the other groups, with the samples distributed in the central region of the graph. MP-30, LP-30, and CK-30 alternately overlap and are distributed on the left side of the figure, while CK, MP, and HP alternately overlap and are distributed on the right side of the figure. Supported by the NCM model and NMDS analysis provides clear insights into changes in bacterial communities during anaerobic fermentation. The results showed that salt stress had significant effects on the microbial composition of fresh and 30-day-old alfalfa silage. This information helps us understand the dynamics of bacterial communities and helps optimize anaerobic fermentation processes to improve silage quality. This study employed the NCM to analyze the impact of Na^+^ and K^+^ concentrations on the assembly mechanisms of bacterial communities in alfalfa silage. The degree of community assembly was assessed by calculating the model’s goodness-of-fit (R^2^).

### The relationships among the ions, microbial community, and characteristics products in alfalfa silage

**Figure 3:**
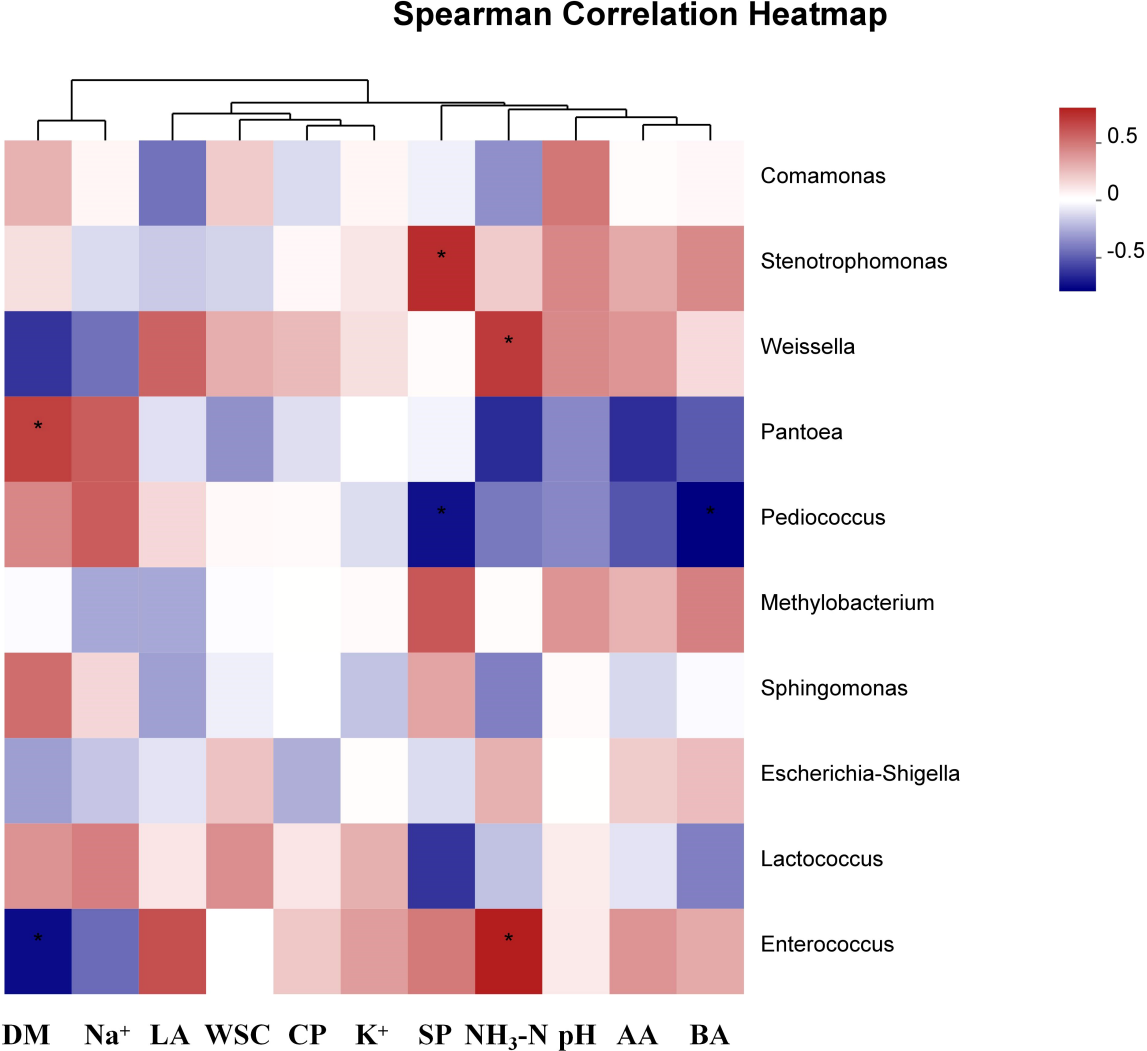
Heatmap of prominent bacterial genera (10 most abundant genera) for alfalfa silage. CK, without salt stress; LP, under light salt stress; MP, under moderate salt stress; HP, under severe salt stress; 30, 30 days of ensiling. *, *P* < 0.05.

The pH of the silage is positively correlated with the abundance of *Comamonas*, *Stenotrophomonas*, and *Weissella*, and negatively correlated with the abundance of *Pantoea* and *Pediococcus* (**Figure 3**). The DM content of the silage is significantly negatively correlated with the abundance of *Weissella* and *Enterococcus* (*P* < 0.01). The WSC content of the silage is positively correlated with the abundance of *Escherichia–Shigella* and *Lactococcus* (*P* < 0.05). The Na^+^ content of the silage is significantly positively correlated with the abundance of *Pantoea*, and significantly negatively correlated with the abundance of *Escherichia–Shigella*, *Weissella*, *Stenotrophomonas*, *Enterococcus*, and *Methylobacterium* (*P* < 0.05). The LA content of the silage is positively correlated with the abundance of *Lactococcus* (*P* < 0.01). The BA content of the silage is positively correlated with the abundance of *Weissella*, *Stenotrophomonas*, *Enterococcus*, *Methylobacterium*, and *Escherichia–Shigella* (*P* < 0.05).

**Figure 4:**
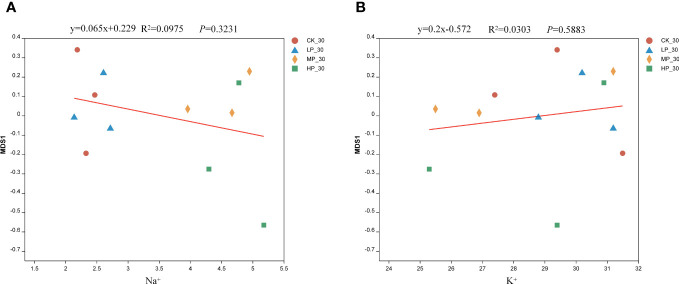
Regression analysis of alfalfa microbial community and sodium ions **(A)** and potassium ion **(B)**. CK, without salt stress; LP, under light salt stress; MP, under moderate salt stress; HP, under severe salt stress; 30, 30 days of ensiling.

The regression equation for Na^+^ and the bacterial community in alfalfa silage is y = 0.065x + 0.229 (**Figure 4A**). As the concentration of Na^+^ increases, the beta diversity of the bacterial community in alfalfa silage follows the order CK-30 > MP-30 > LP-30 > HP-30. This study demonstrates a significant positive correlation between Na^+^ and the beta diversity of the bacterial community in alfalfa silage, indicating that Na^+^ has an impact on the structure of the bacterial community. It can be observed that the regression equation for K^+^ and the bacterial community in alfalfa silage is y = 0.2x − 0.572 (**Figure 4B**). The beta diversity of the bacterial community in alfalfa silage follows the order CK-30 > MP-30 > LP-30 > HP-30. These findings highlight the influence of endogenous Na^+^ and K^+^ in alfalfa on the structure of the bacterial community in salinity–alkalinity soil. They further confirm the impact of endogenous Na^+^ and K^+^ in plants on the stability and quality of bacterial communities during silage fermentation.

### Co-occurrence network analysis of bacterial communities

**Figure 5:**
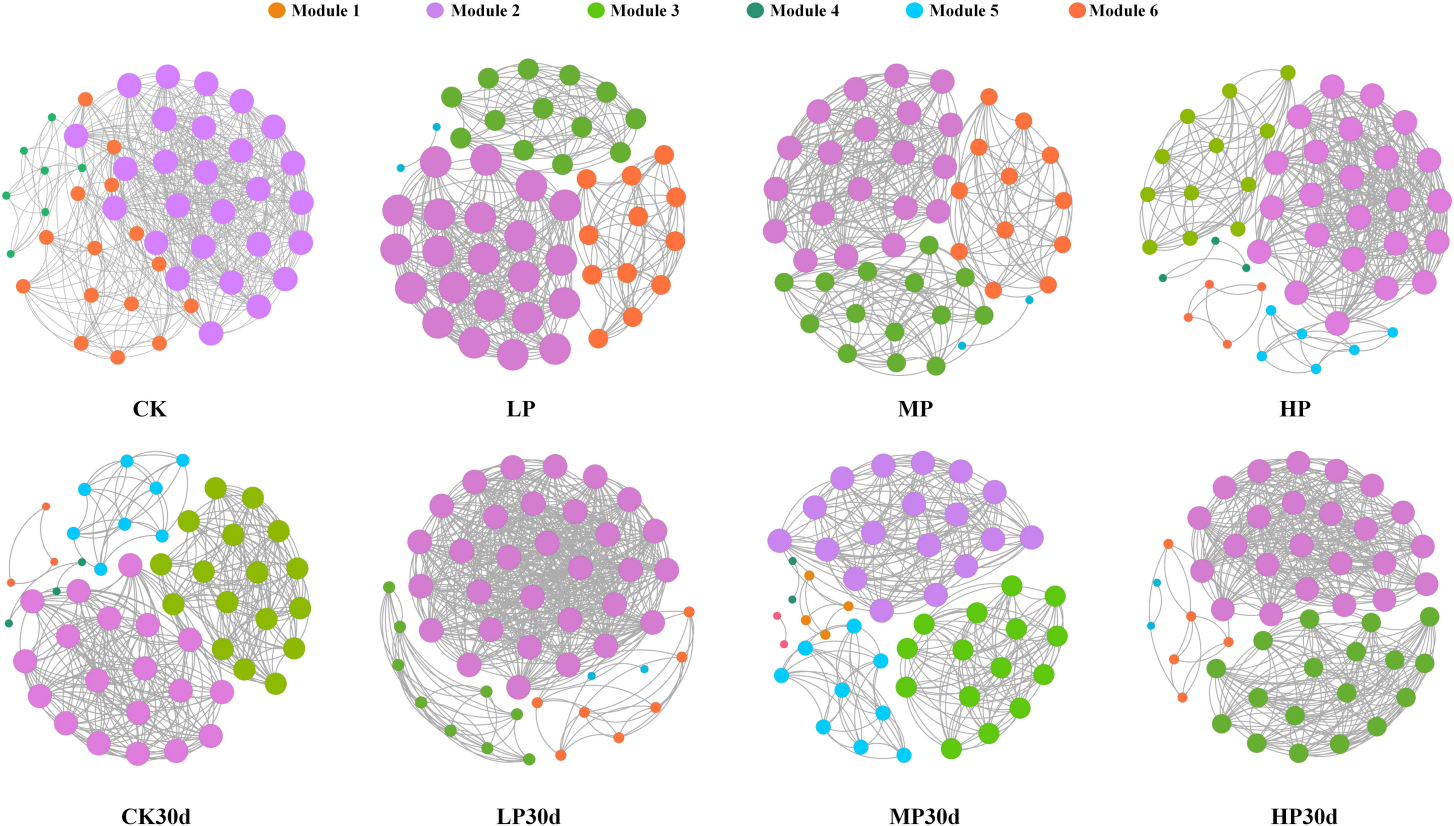
Differences of co-occurrence network visualization in each group. CK, without salt stress; LP, under light salt stress; MP, under moderate salt stress; HP, under severe salt stress; 30, 30 days of ensiling.

Significant changes in microbial populations within silage occurred under different salt stress levels, with evident interactions among different genera (**Figure 5**). Additionally, the corresponding network analysis revealed that lower salt stress levels resulted in a more complex and interconnected microbial network compared to higher stress levels. Specifically, there was an increase in the number of positive correlations between different bacterial genera under low salt stress, indicating a more diverse and stable microbial community. In contrast, the microbial network complexity observed in the MP group was relatively low, with fewer positive correlations among different genera. These findings suggest that the LP group may induce synergistic effects on the microbial community within the silage, leading to increased diversity and stability within the microbial ecosystem. Furthermore, after 30 days of ensiling, the LP group exhibited a relatively complex microbial community. Overall, based on the topological metrics, the LP group displayed the most complex network with significant microbial interactions.

## Discussion

### The chemical composition of fresh alfalfa

Salt stress affects the normal growth and development of plants and affects the chemical composition of plants (Monroy and Ericsson, 2023). Most plants are sensitive to salt, and alfalfa is no exception. When plants are subjected to salt stress, both their growth and chemical composition are affected. Significant differences exist in SP content among different groups. Compared to the CK, the low-concentration salt stress groups (LP and MP) exhibited a significant increase in SP content. Among them, the MP group had the highest SP content, reaching 103.7 g/kg. As salt concentration increased, the SP content in the HP group significantly decreased, consistent with the findings of Horchani et al. (2023), who demonstrated a decrease in SP content under high salt stress conditions. Carbohydrates are typically categorized as non-structural and structural carbohydrates. Non-structural carbohydrates are found within plant cells and mainly include sugars, starch, organic acids, and other storage carbohydrates (Kristin et al., 2023). Under salt stress, plants generally increase their carbohydrate levels, such as sugars and starch, to mitigate the stress (Shao et al., 2022). The WSC content in the MP group was significantly higher than that in the HP group. This indicated that as salt stress intensity increased, the WSC content gradually decreased, suggesting that salt stress was an important factor influencing the chemical composition of alfalfa.

### Fermentation characteristics and chemical composition of alfalfa silage

The pH of silage is an important indicator for evaluating the fermentation effect, and a pH of 4.2 is considered as the benchmark for high-quality silage (He et al., 2021). With increasing salt concentration, the pH of the four silage groups continuously decreased. The results of the study showed that the pH of the LP and MP groups was significantly lower than that of the CK and HP groups from day 1 to day 30. At day 30 of silage fermentation, the HP group had the highest pH value of 5.13, while the MP group had the lowest pH value of 4.93. These results indicate that higher pH values are associated with poorer fermentation quality. The variation in pH may be attributed to different salt concentrations in alfalfa or differences in microbial populations during silage fermentation. The significant differences in the chemical composition of alfalfa samples and silage fermentation characteristics are closely related. The lower pH and higher LA concentration in the MP silage group are due to the rapid metabolism of WSC into LA by LAB, resulting in a decrease in pH and stability of the silage within a short period (Wang et al., 2021).

During the 30-day fermentation period, *Weissella*, *Enterobacter*, and *Pseudomonas* were detected. *Weissella* and *Enterobacter* not only consumed a large amount of WSC content but also exhibited low utilization efficiency of WSC (Blajman et al., 2020). Therefore, with increasing salt concentration and fermentation days, the WSC content in all groups continuously decreased. BA, LA, and AA reflect the efficiency of silage fermentation or secondary fermentation. In addition, as the LA content continuously increased, the BA content gradually increased as well, indicating that the amount of BA depends on the amount of LA. This may be caused by secondary fermentation by heterofermentative LAB and yeast (Qiang et al., 2021a).

The presence of ammonia nitrogen (NH_3_-N) during the ensiling process is an important indicator of the protein hydrolysis (He et al., 2020). The inhibitory effects of MP and HP on ammonia accumulation suggest enhanced preservation of protein during the ensiling process. In the ensiling process, protein undergoes extensive degradation and deamination of amino acids (Bachmann et al., 2020), and a typical reason for ammonia nitrogen accumulation is protein hydrolysis enzymes (Tian et al., 2022). After the fermentation of alfalfa, the SP content increased from 66.7–104.3 g/kg to 124.3–150.3 g/kg, which may be related to protein hydrolysis during the fermentation. The degradation of macromolecular proteins into small molecular proteins with water-soluble characteristics could be one of the reasons for the increase in SP content (Liu et al., 2021).

### Microbial community of alfalfa silage

In this study, LAB, *Escherichia coli*, and some aerobic bacteria were found to dominate in all alfalfa silages. These findings are consistent with previous research reports on alfalfa silage and even corn silage (Guo et al., 2020). Mold was observed in the CK and LP groups on day 1 of ensiling, but it disappeared as the duration of ensiling and salt concentration increased. This may be attributed to the metabolites produced by LAB, which inhibit the growth of harmful bacteria such as Clostridium botulinum and mold (Qixuan et al., 2021). LAB and *Escherichia coli* were the most abundant microbial types during ensiling. Nazar et al. (2020) reported a transition in the bacterial community from Proteobacteria to Firmicutes, and they found that anaerobic and acidic conditions favored the growth of Firmicutes. Additionally, the quantity of *Escherichia coli* decreased regularly with increasing salt stress.

Biological and abiotic stresses are closely related to the growth and development of alfalfa, with salt stress being a major abiotic stress factor affecting yield and nutritional quality (Shao et al., 2022). Microbes play a significant role in regulating plant growth, stress resistance, and disease resistance (Wang et al., 2021), and the structure of microbial communities is influenced by plant species and growth stages. After experiencing biotic and abiotic stresses, plants can alleviate the harm caused by stress by adjusting the structure of the microbial community (Afridi et al., 2022). Bouzroud et al. (2023) found that plants without symbiotic microorganisms are more susceptible to diseases and less likely to survive in natural environments.

The genus and quantities of microorganisms are closely related to the nutritional and fermentation quality of alfalfa silage (Qiang et al., 2021a). In the MP group, there was a strong positive correlation between general aerobic bacteria, *Escherichia coli*, and CP, WSC, ADF, and NDF. From the perspective of the relationship between the main nutrients and microorganisms in alfalfa silage from saline–alkaline land, a higher quantity of LAB is associated with better nutritional quality of alfalfa, while higher quantities of *Escherichia coli*, mold, and general aerobic bacteria are unfavorable for the nutritional preservation of alfalfa silage from saline–alkaline land. In moderately saline–alkaline land alfalfa silage, there was a negative correlation between general aerobic bacteria, *Escherichia coli*, mold, and SP content. This is consistent with Chauhan et al. (2023) previous research. Based on the above analysis, it can be concluded that LAB is the key factor influencing the nutrition of alfalfa silage from different saline–alkaline lands. The species and quantity of LAB in alfalfa silage raw materials directly determine the quality of alfalfa silage and are crucial for the success of silage fermentation (Qiang et al., 2021b). LAB can establish a dominant microbial population during the silage process when their quantity exceeds 10^5^ Log_10_ cfu/g, meeting the requirements for silage (Zhu et al., 2022). In this study, the LAB content in alfalfa silage raw materials under different salt stress ranged from 6.41 Log_10_ cfu/g to 6.59 Log_10_ cfu/g, meeting the requirements for direct silage. In saline–alkaline land alfalfa silage, the quantity of LAB increased with the duration of silage, and the quantity of LAB in the 30-day silage fermentation was significantly higher than that in the silage raw materials. In this study, the quantity of LAB dominated after 3 days of silage, and no molds were found in all groups after 3 days of silage, while no *Escherichia coli* was found in all groups after 7 days of silage. This is consistent with the findings of Dong et al. who reported that in the later stages of silage fermentation, the accumulation of LA produced by LAB and the decrease in pH inhibit the growth of *Escherichia coli* and mold (Dong et al., 2020).

The LAB played a crucial role in the process of ensiling, while soluble carbohydrates serve as fermentation substrates necessary for normal microbial metabolism in silage feed, it is also the main factor affecting the fermentation quality of silage (Stirling et al., 2022). As the ensiling process progresses, soluble carbohydrates are metabolizes by LAB to produce organic acids, resulting in a decrease in soluble carbohydrate content (Luc and Frédéric, 2020). In this study, pH rapidly decreased starting from the 5th day of fermentation, and this downward trend in pH was closely related to the increase in the population of LAB on the 5th day of fermentation. Meanwhile, the average lactate content gradually increased. The low pH and high lactate content in the MP and HP groups can be attributed to the LAB, which rapidly metabolize soluble carbohydrates into LA through LA fermentation (Nazar et al., 2020). The increased LAB species include Lactobacillus, *Enterococcus*, *Enterobacter*, and *Streptococcus*. This is consistent with the findings of Mariele et al., where LA production by LAB led to a decrease in the pH of the silage feed (Agarussi et al., 2019). The results of this study indicate that the pH variation may be attributed to the differences in the adherent bacterial community of alfalfa from different saline–alkaline lands, which in turn have an impact on the differential lactate and acetate contents observed in this study. These findings further confirm the significant relationship between chemical composition and microbial communities. Understanding the composition and distribution of microorganisms is of great importance for improving silage feed quality, promoting the development of the forage processing industry, and conserving and utilizing microbial resources.

Na^+^ and K^+^ were likely the cause of microbial community changes in this study, as plant-associated microorganisms exhibit different reactions to varying salt stress levels. In our investigation, both *Lactococcus* and *Pantoea* showed a gradual increase in abundance with increasing salt stress, particularly dominating in the HP-30 group. An anaerobic environment, compared to fresh samples, brings about alterations in the microbial habitat, suppressing aerobic microorganisms during anaerobic fermentation and consequently resulting in noteworthy differences between fresh and ensiled microbial communities (Nazar et al., 2020). Bacterial diversity and richness of CK, LP, MP, and HP groups all decreased after 30 days of fermentation. Similarly, Jie et al. (2020) reported a decrease in bacterial diversity in silage due to the increased abundance of dominant genera such as *Lactococcus* and *Enterococcus*. By the 30th day of ensiling, *Enterococcus*, *Lactococcus*, *Escherichia*–*Shigella*, and *Sphingomonas* were the dominant genera in all groups. The growth and proliferation of *Pantoea*, *Pseudomonas*, and *Methylobacterium* were inhibited by the lower pH, while the relative abundance of *Pantoea* gradually declined with increasing salt stress. Under anaerobic conditions, *Enterococcus* dominated in CK-30 (37.8%) and LP-30 (64.1%). Similar to other LAB members, *Enterococcus* exhibits the ability to survive, resist, and proliferate under adverse conditions, including low and high pH levels, high temperature, and osmotic stress (Acciarri et al., 2023).

In this study, A relatively low R^2^ value (0.5797) was observed, suggesting that the assembly of bacterial communities in alfalfa silage aligns more closely with the neutral model, indicating a greater susceptibility to deterministic processes and less influence from stochastic processes. As the content of Na^+^ and K^+^ increased, the richness of the bacterial community gradually decreased. This may be attributed to the increase in taxonomic groups within the bacterial community under salt stress, which aligns with the findings of Meng et al. (2019). Their research revealed that the bacterial community exhibits a highly active and sensitive response to Na^+^ and K^+^. The abundance of *Escherichia–Shigella* and *Comamonas* was negatively correlated with CP in silage, while the abundance of *Enterococcus* and *Lactococcus* showed a positive correlation. With increasing salt stress and ensiling duration, the CP content continued to rise. This may be due to the higher sodium ion concentration in alfalfa under salt stress, which inhibits bacterial proliferation and subsequently reduces protein breakdown (Hong et al., 2023).

## Conclusion

The study presented herein examined the presence of endogenous Na^+^ and K^+^ in plants significantly impacts the quality of alfalfa silage and the stability of bacterial communities during fermentation. The impact of endogenous sodium and potassium ions on alfalfa silage is twofold. Increased salt stress leads to changes in microbial composition, with *Lactococcus* and *Pantoea* exhibiting a gradual increase in abundance, particularly in the highly salt-stressed group. Moreover, low pH inhibits the growth and reproduction of certain bacterial genera, including *Pantoea* and *Pediococcus*. The abundance of *Escherichia*–*Shigella* negatively correlates with CP, whereas *Enterococcus* and *Lactococcus* exhibit a positive correlation. The accumulation of endogenous ions in alfalfa under salt stress suppresses bacterial proliferation, thereby reducing protein degradation during fermentation. To harness the influence of endogenous sodium and potassium ions on alfalfa silage, it is imperative to develop tailored strategies aimed at optimizing the fermentation process. These valuable findings provide a solid foundation for the refinement of techniques and approaches in silage production and preservation, ultimately enhancing overall silage quality and nutritional value.

## Data availability statement

The datasets presented in this study can be found in online repositories. The names of the repository/repositories and accession number(s) can be found below: https://www.ncbi.nlm.nih.gov/, PRJNA753242.

## Author contributions

JS: Writing – original draft. GZ: Data curation, Writing – review & editing. WK: Data curation, Writing – review & editing. YP: Software, Writing – review & editing. MH: Software, Writing – review & editing. CC: Methodology, Writing – review & editing. DS: Formal analysis, Writing – review & editing. ML: Conceptualization, Writing – review & editing. YL: Conceptualization, Writing – review & editing. QL: Conceptualization, Writing – review & editing.
